# Progress of carbon-based electrocatalysts for flexible zinc-air batteries in the past 5 years: recent strategies for design, synthesis and performance optimization

**DOI:** 10.1186/s11671-021-03548-5

**Published:** 2021-05-25

**Authors:** Yuan Qin, Zihao Ou, Chuanlan Xu, Zubang Zhang, Junjie Yi, Ying Jiang, Jinyan Wu, Chaozhong Guo, Yujun Si, Tiantao Zhao

**Affiliations:** 1 College of Chemistry and Chemical Engineering, Chongqing University of Technology, Chongqing, 400054 China; 2 Chongqing Key Laboratory of Materials Surface and Interface Science, Chongqing University of Arts and Sciences, Chongqing, 402160 China; 3 College of Chemistry and Chemical Engineering, Chongqing University, Chongqing, 401331 China; 4 College of Chemistry and Materials Science, Sichuan University of Science and Engineering, Zigong, 643000 China

**Keywords:** Flexible zinc-air batteries, Carbon-based electrocatalysts, Electrocatalytic mechanism, Air cathode

## Abstract

The increasing popularity of wearable electronic devices has led to the rapid development of flexible energy conversion systems. Flexible rechargeable zinc-air batteries (ZABs) with high theoretical energy densities demonstrate significant potential as next-generation flexible energy devices that can be applied in wearable electronic products. The design of highly efficient and air-stable cathodes that can electrochemically catalyze both the oxygen reduction reaction (ORR) and oxygen evolution reaction (OER) are highly desirable but challenging. Flexible carbon-based catalysts for ORR/OER catalysis can be broadly categorized into two types: (i) self-supporting catalysts based on the in situ modification of flexible substrates; (ii) non-self-supporting catalysts based on surface coatings of flexible substrates. Methods used to optimize the catalytic performance include doping with atoms and regulation of the electronic structure and coordination environment. This review summarizes the most recently proposed strategies for the synthesis of designer carbon-based electrocatalysts and the optimization of their electrocatalytic performances in air electrodes. And we significantly focus on the analysis of the inherent active sites and their electrocatalytic mechanisms when applied as flexible ZABs catalysts. The findings of this review can assist in the design of more valuable carbon-based air electrodes and their corresponding flexible ZABs for application in wearable electronic devices.

## Introduction

The current excessive usage of non-renewable energy has raised concerns regarding the energy crisis. Therefore, to alleviate the current energy shortage, more efficient and environmentally friendly power supply devices need to be established. In addition, the emergence and popularization of stretchable, foldable and bendable wearable electronic devices have instigated the rapid growth and development of flexible energy storage systems [1–3]. Zinc-air batteries (ZABs) exhibit a theoretical energy density of 1086 Wh kg^−1^, which is approximately five times that of the widely used rechargeable lithium-ion batteries. Moreover, zinc has the advantages of abundant reserves and wide availability [4, 5]. Typical ZABs utilize zinc as the negative electrode, oxygen as the positive electrode and potassium hydroxide as the electrolyte. Owing to the introduction of highly stable zinc anodes and water-based electrolytes, ZABs are non-toxic, environmentally friendly and safe and have received widespread attention as promising energy storage systems [6]. The basic working principle of ZABs involves an electrochemical reaction between zinc on the negative electrode of the battery and the OH^−^ in the electrolyte solution resulting in the release of electrons. Simultaneously, the catalysts in the gas diffusion electrode or air cathode reaction layer come in contact with the electrolyte and the oxygen in the air, and then the charge transfer occurs. During the operation of rechargeable ZABs, the conversion between oxygen and water occurs on the air electrode; this includes the ORR and OER, both of which are multi-electron recombination processes. The specific reactions that occur in alkaline solutions are as follows:

1 $${\text{ORR}}:\,{\text{O}}_{2} \left( {\text{g}} \right) + 2{\text{H}}_{2} {\text{O}}\,\left( {\text{l}} \right) + 4{\text{e}}^{ - } \to 4{\text{OH}}^{ - }$$

2 $${\text{OER}}:\,4{\text{OH}}^{ - } \to {\text{O}}_{2} \left( {\text{g}} \right) + 2{\text{H}}_{2} {\text{O}} \left( {\text{l}} \right) + 4{\text{e}}^{ - }$$

Various kinetic models have been developed to understand the reaction pathways involved during the ORR. The first model, developed by Damjanovic et al. [7, 8], involves the formation of hydrogen peroxide in a reaction pathway parallel to that in which O_2_ is reduced to water without the formation of hydrogen peroxide as an intermediate. This is schematically represented by Eq. 1 and 2.

1 $${\text{O}}_{{2}} \to ^{{{\text{I}}_{{1}} }} {\text{H}}_{{2}} {\text{O }}$$

2 $$\begin{array}{*{20}c} {{\text{O}}_{{2}} \mathop{\longrightarrow}\limits^{{{\text{I}}_{{2}} }}{\text{H}}_{{2}} {\text{O}}_{{2}} \mathop{\longrightarrow}\limits^{{{\text{I}}_{{3}} }}{\text{H}}_{{2}} {\text{O}}} \\ { \downarrow {\text{I}}_{4} } \\ {{\text{to}}\,{\text{solution}}\,{\text{and}}\,{\text{ring}}\,{\text{electrode}}} \\ \end{array}$$

Hydrogen peroxide, formed as a reaction intermediate in pathway 2, is partially reduced at the same disk electrode as water and partially transferred from the disk electrode to the solution and ring electrode by convective diffusion. I_1_, I_2_ and I_3_ represent the respective currents. I_4_ represents the rate at which hydrogen peroxide diffuses away from the disk electrode as a current. However, the generated peroxide intermediate is unstable, which may complicate the reaction process, damage the electrolyte membrane and reduce the activity of the catalyst, as well as the output voltage and energy conversion rate of the fuel cell [9]. Therefore, the direct 4e^–^ pathway (Eq. 1) is considered to be the ideal pathway for the ORR, as it has a higher output voltage and energy conversion than the 2e^–^ pathway (Eq. 2).

Because a considerable over-potential of the ORR is required to overcome the energy barriers associated with multi-step electron transfer [10], the main challenge faced in developing flexible rechargeable ZABs that can be applied on a large scale is the use of air cathodes in the ZABs that exhibit excessive potential [11] and poor oxygen reversibility caused by the slow ORR and OER during charge and discharge [12]. The Pt/C electrodes demonstrate the best catalytic performance for the ORR, whereas IrO_2_/RuO_2_ demonstrates an excellent catalytic performance in the OER. However, these catalysts also suffer from several drawbacks, such as scarce reserves, high cost, single catalytic activity and poor stability, which severely hamper their application on a large scale [13]. Thus, the development of a catalyst with excellent bifunctional ORR/OER catalytic performances that is affordable is vital for the commercialization of flexible ZABs. Non-noble metals, particularly transition metals, have attracted widespread attention owing to their high activity and excellent thermal stability. In addition, carbon-based catalysts possess significant advantages, including structural flexibility, excellent electrical conductivity, good chemical and thermal stability and simple chemical functionalization, in addition to being lightweight. Thus, they are regarded as promising candidate materials for use in wearable electronic products. There exist several excellent CC (carbon cloth)-based air cathodes; however, one of the main challenges is the identification of materials with excellent conductivity that can uniformly grow on CC. If the materials grow in a disorderly fashion on the CC, the number of active sites on the catalyst are reduced. Traditional ZABs use an aqueous solution as the electrolyte, which cannot meet the requirements of solid-state flexible ZABs. Thus, most solid-state ZABs that exhibit excellent performances use gel electrolytes to conduct electricity, such as polyvinyl alcohol, polyethylene oxide (PEO), polyacrylamide (PAM) and polyacrylic acid (PAA). These gel electrolytes are increasingly used in ZABs, as they possess strong plasticity and good conductivity. Specifically, sodium polyacrylate is stable in the practical application of water-based gel electrolytes, owing to its buffering effect in alkaline electrolytes. [1]

In recent years, the number of studies on flexible ZABs has increased, providing some theoretical basis for the practical production and application of flexible ZABs. Although Zhu et al. published a detailed review of one-dimensional batteries [14] and Shi et al. introduced bifunctional catalysts in detail [15], the recent progress, particularly in the past five years, of flexible ZABs containing carbon-based catalysts has not been reported. Therefore, this work summarizes the strategies for the synthesis of carbon-based catalysts and the optimization of their electrocatalytic performances in air electrodes, with a significant focus on the analysis of their inherent active sites and their electrocatalytic mechanism when applied as flexible ZABs catalysts.

### Designer carbon-based electrocatalysts

Carbon-based materials are widely used in ZABs catalysts owing to their excellent properties. These materials are mainly graphene-based materials (including functionalized graphene and graphene profiles); however, graphite, fullerene and carbon nanotubes (CNTs) are also used [16]. Nevertheless, carbon-based materials still suffer from many defects during the practical application of ZABs. Thus, it is necessary to optimize the treatment applied to carbon materials. An example of this is N-doped porous carbon materials, which exhibit exceptional bifunctional electrocatalytic performances in the ORR and OER [17, 18]. Among the variety of methods used to modify carbon materials, doping with single atoms, such as N, P and S, can significantly improve the catalyst activity. In view of this, some researchers have used N and P co-doping and found that the co-doped catalyst has excellent activity. In addition, other methods, such as single metal doping, bimetal doping and nanomaterials, have a definite positive effect on the catalyst performance optimization of carbon materials. However, methods to develop high-performance electrocatalysts for ZABs have been scarcely explored. Studies have shown that modifying carbon materials, such as graphene and CNTs, through doping can optimize the surface properties of the carbon materials. The most typical strategy to optimize the catalyst performance is to combine heteroatom-doped carbon nanomaterials with transition metal-based material (oxides, chalcogenides such as Ni-based sulfides, etc. [19–21], transition metal phosphides (TMP [22]) and nitride) composites. As ZABs electrocatalysts still have numerous shortcomings, it is vital to optimize their catalytic structure. Currently, electronic structure adjustment, oxygen defects, metal–oxygen bonds, interface strain and atomic doping have been widely used in the design of ZABs catalysts.

### Growing high-efficiency catalysts on flexible electrodes

The flexible electrode is represented by the gas diffusion layer in flexible ZABs. The air cathode is formed by directly growing a high-efficiency catalyst on the flexible electrode, which has the advantages of being self-supporting and having a large electrode contact area and strong foldability. Self-supporting indicates that no non-conductive adhesive is required, thereby avoiding the deterioration of the electrode performance and loss of the catalyst during repeated deformation of the electrode. Furthermore, the reduction in active sites and increase in interface impedance is avoided by using non-conductive adhesives. Growth of the catalyst on the expandable electrode can be combined with other methods, such as electrodeposition, the hydrothermal method and room temperature vulcanization. Commonly used flexible electrodes include nitrogen-doped carbon foam, carbon fiber cloth, carbon paper and carbon felt, which possess excellent electrical conductivity.

### Growth of high-efficiency catalysts on carbon fiber cloth

**Fig. 1:**
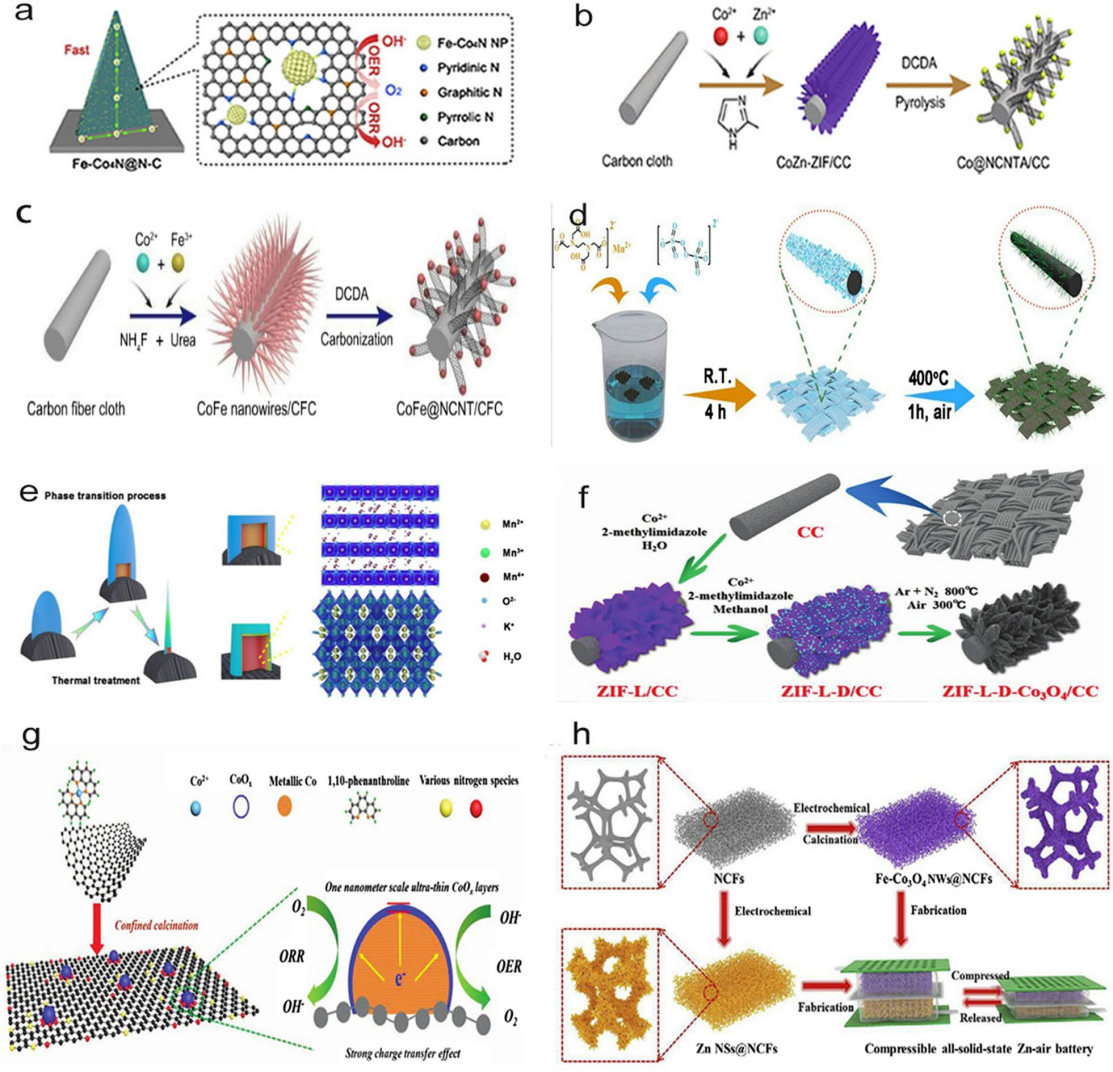
**a** Schematic of the Fe-Co_4_N@N–C nanosheet grown on CC for application in the bifunctional oxygen reaction [56]. **b** Schematic depicting the synthetic process for Co@NCNTAs [85]. **c** Schematic of the synthetic process for CoFe@NCNT/CFC [98]. **d** Schematic of the preparation of MnO_x_-CC-400 [2]. **e** Proposed phase transition of manganese oxide on CC via a thermal treatment [2]. **f** Schematic of the ZIF-L-D-Co_3_O_4_/CC formation process [24]. **g** Schematic of a 1 nm-CoO_*x*_ layer on the metallic substrate of Co/N-RGO [23]. **h** Schematic of the fabrication processes for the squeezable and rechargeable all-solid-state ZABs [27]

Carbon fiber cloth, a woven fabric constructed from carbon fibers, is the most commonly used flexible substrate material. Growing high-efficiency catalysts directly on carbon fiber cloth is a simple and effective method, which can be achieved through solution reactions (Fig. 1a–c), electrodeposition and a combination of other methods, such as confined space, heat treatment [2] (Fig. 1d, e), carbonization-oxidation CC and ligand-assisted calcination (preparation of an ultrathin CoO_X_ layer [23]). An example of carbonization-oxidation includes the growth of different crystals structures, morphologies and particle sizes of 3D and 2D cobalt-based MOFs on CC for the preparation of a binder-free cathode, followed by anchoring of the layered Co_3_O_4_ nanoparticles in nitrogen-doped carbon nano-arrays [24]. The nanofiber network is rooted on CC in a nitrogen atmosphere to obtain a bifunctional air cathode with an excellent catalytic performance and remarkable flexibility [25]. Although the electrodeposition method has been widely used to prepare electrode materials, owing to the inherently poor conductivity of Co_3_O_4_, conventional electrodeposition methods exhibit limitations in forming a Co_3_O_4_ layer with a large contact area on a conductive carrier. Co_3_O_4_ can be grown in situ on the carbon fiber cloth to form a uniformly grown ultrathin Co_3_O_4_ layer. In particular, the ultrathin Co_3_O_4_ layers have a maximum contact area on the conductive support, facilitating rapid electron transport and preventing aggregation of the ultrathin layers during the electrode preparation process [26]. Moreover, Co_3_O_4_ can be converted into a nano-microarray with a layered structure [24], as depicted in Fig. 1f. This ultrathin cobalt oxide layer can also be used as an electrocatalyst in ZABs [23], as depicted in Fig. 1g.

### Growth of efficient catalysts on self-made foam materials

**Fig. 2:**
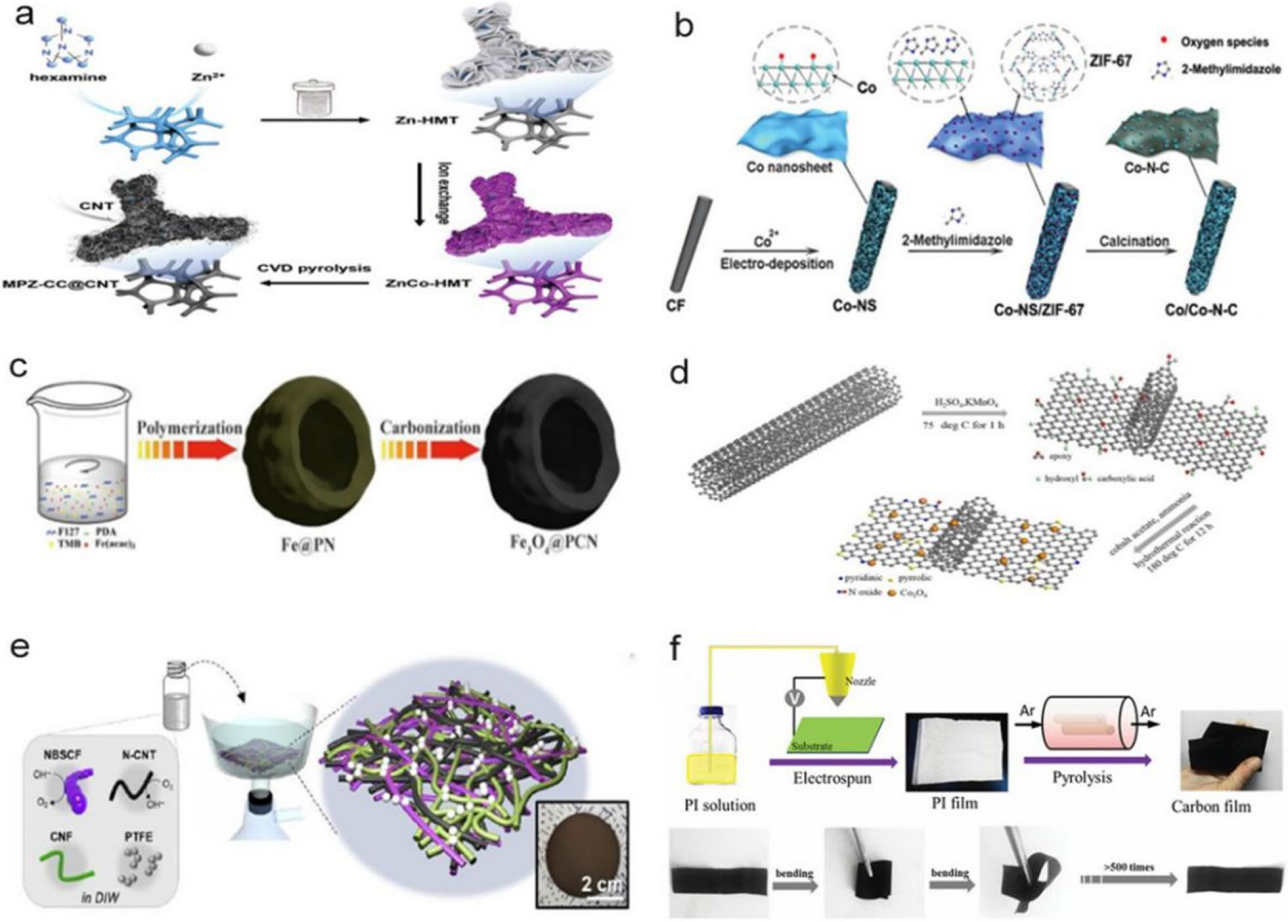
**a** Schematic of the preparation process for MPZ-CC@CNT [28]. **b** Synthesis of the Co/Co–N-C catalyst [29]. **c** Schematic of the preparation of the Fe_3_O_4_@PCN catalysts [40]. **d** Scheme depicting the Co_3_O_4_/N-p-MCNTs composite catalyst synthesis [41]. **e** Scheme of the fabrication procedure for the MH paper air cathode, along with its photograph [43]. **f** Schematic representation of the fabrication procedure for the NCNF and photographs of the resultant flexible NCNF [44]

Self-made foam materials mainly refer to carbon foam and nickel foam. Pan et al. used a melamine sponge annealed at a temperature of 800 °C to form a flexible foamed carbon material, which was then used as a working electrode to electrodeposit the precursor for Fe-Co_3_O_4_NWS@NCFs via a scalable electrodeposition method. Consequently, the flexible foamed carbon material could be applied in all-solid sponge batteries [27], as shown in Fig. 1h. The nickel foam material is a type of reticulated metal sponge. Jiang et al. indicated that growing catalyst electrodes in situ on a flexible substrate would cause disorder and form dense irregular areas, which would reduce the catalytic activity. Therefore, they constructed an ordered multidimensional array of 1D CNTs decorated with 0D cobalt nanoparticles (called MPZ-CC@CNT) and 2D carbon nano-ridges on a nickel foam material. During the pyrolysis of the 2D ZnCo bimetallic coordination framework, CNTs containing a high content of N-doping were grown in situ from the highly dispersed cobalt, thereby forming an open and ordered array [28], as depicted in Fig. 2a.

### Synthesis of high-efficiency catalysts on carbon felt

Carbon felt is a non-woven fabric. Yu et al. [29] developed a novel strategy for synthesizing Co–N-C nanosheets supported on carbon felt (Co/Co–N-C), containing Co nano-islands with a 3D-layered structure, as illustrated in Fig. 2b. This unusual structure results in good contact between the Co nano-islands and Co–N-C nanosheets. Additionally, the coexistence of Co^0^ and Co^2+^ enhanced the electrocatalytic performance of the bifunctional (ORR/OER) catalyst. Therefore, the overall unique layered structure can further promote effective electron/ion transport in ORR and OER [29]. In addition to growing 3D nanoelectrodes on carbon felt, a nanoscale ultrathin cobalt oxide (CoOx) layer can also be fabricated on carbon felt (i.e., a metal Co/N-doped graphene substrate) [23]. This ultrathin structure provides favorable conditions for application in ZABs.

### Synthesis of high-efficiency catalysts on carbon paper

Carbon fiber paper (CFP) is composed of carbon fiber and produced through the papermaking process. CFP can be used to improve the ORR and OER performances of non-metallic electrocatalysts, such as g- C_3_N_4_. G-C_3_N_4_ has a high nitrogen content and can thus provide a sufficient number of active sites for electrocatalytic reactions and reduce the ORR potential [30]. However, its electrocatalytic performance is significantly limited owing to its non-conductivity leading to a poor electron transfer ability [30]. G-C_3_N_4_ with different morphologies can be prepared using different heat treatment methods [30]. The ORR and OER performances of the catalyst can be optimized by applying treatments to g-C_3_N_4_, such as 1) synthesizing a high-efficiency catalyst using g-C_3_N_4_ as a template [31, 32], 2) applying a g-C_3_N_4_-assisted pyrolysis strategy [33–35], or 3) introducing g-C_3_N_4_ into carbon. Phosphorus-doped g-C_3_N_4_ can directly grow on CFP, which can be designed as a flexible oxygen electrode. This is the first non-metallic ORR/OER bifunctional electrocatalyst formed by the combination of flower-like Pg-C_3_N_4_ composed of thin nanosheets of g-C_3_N_4_ and CFP. The electrocatalyst contains a 3D hybrid network with a high N content and a large amount of P-doping, which produces an excellent ORR and OER activity and durability [36], and a good charge and discharge performance, even in the case of bending deformation.

### Synthesis of high-efficiency catalysts in confined spaces

The synthesis of high-efficiency catalysts in confined spaces can increase doping efficiency, reduce heat loss and increase mesoporous properties, thereby improving their ORR performance. The confined spaces can be molecular sieve nanochannels, such as montmorillonite, 2–4-6 tripyridyl triazine, CNTs, carbon nanosheets and doped carbon layers. Numerous experiments have also demonstrated that catalysts prepared in confined spaces exhibit an improved catalytic effect. Doping transition elements and non-metallic elements in confined spaces can significantly enhance catalytic efficiency. N and S co-doping [13], N and P co-doping [12], two-dimensional nitrogen doping [37, 38], etc. have been reported. Furthermore, it has been established that if a plasma-assisted strategy is used for doping in a confined space, the etching effect of the plasma can endow porosity to the confined space, thereby exposing more active sites, which is conducive to the long-term durability and effective electron transport of the electrocatalyst [39]. Zhang et al. used a soft membrane method to prepare Fe_3_O_4_ wrapped in a porous carbon nano-bowl, demonstrating excellent catalytic performance and long-term durability [40], as shown in Fig. 2c. The Co_3_O_4_ nanoparticles were anchored on the partially exfoliated multi-walled CNTs doped with nitrogen, resulting in an outstanding catalytic performance [41], as shown in Fig. 2d.

### Combination of high-efficiency flexible catalysts

A combined catalyst comprises a single functional catalyst composed of the same material in different shapes or a bifunctional catalyst composed of different materials. Xu et al. achieved a single-function ORR air cathode by simultaneously designing aligned, cross-stacked and porous CNT sheets, where the CNT sheets functioned as a gas diffusion layer, a catalyst layer and a current collector, and synthesized a new fibrous, flexible and stretchable ZABs [42]. The air cathode catalyst in flexible ZABs typically exhibits insufficient ORR/OER catalytic activity and requires harsh synthesis conditions, including high temperatures/high pressures and acid (or alkaline) solutions. In addition to the problems of bifunctional air catalysts, the mechanical properties of the air cathode strongly depend on the mechanical properties of its substrate, resulting in a lack of shape diversity and deformability in the air cathode sheet. On this basis, Lee et al. used a combined high-efficiency flexible catalyst for the OER and nitrogen-doped CNTs for the ORR [43], as shown in Fig. 2e. The monolithic hetero-nano-mat paper air cathode comprises a 1D bifunctional catalyst mixture, cellulose nanofibers and polytetrafluoroethylene nanoparticles, with no need for conventional current collectors and gas diffusion layers [43]. Zhang et al. synthesized a new type of NiCo_2_O_4_/N-doped carbon nano-mesh bifunctional electrocatalyst composed of hollow NiCo_2_O_4_ nanospheres and N-doped carbon nano-mesh [40]. The bifunctional electrocatalyst was synthesized through a liquid-phase synthesis and subsequently heat treated, after which it was assembled into a battery.

### Home-made flexible cathode film

Liu et al. prepared a nanoporous carbon nanofiber film (NCNF) by pyrolyzing an electrospun polyimide (PI) film under an Ar atmosphere. As shown in Fig. 2f, the NCNF possesses flexibility and high mechanical and tensile strength. The tensile strength of the NCNF is 1.89 MPa, and the tensile modulus is 0.31 GPa. NCNF exhibits a 3D nanoporous carbon network structure and a large specific surface area, which can provide short and fast electron/ion paths and abundant gas diffusion channels. More importantly, the electrode design also has the advantages of eliminating polymer binders and simplifying the manufacturing process, minimizing battery size and cost. The flexible all-solid rechargeable ZABs containing the NCNF air cathode exhibits a high discharge voltage (~ 1.0 V @ 2 mA cm ^−2^), low charging voltage (~ 1.8 V @ 2 mA cm ^−2^), high energy density of 378 Wh kg^−1^ and excellent mechanical and cyclic stability. These results suggest the possibility of large-scale applications of the ZABs [44].

#### Optimization strategies for ZABs catalyst performance

In the past few years, research on inexpensive and highly efficient electrocatalysts for ORR and OER has developed rapidly. Although there are many controversies regarding the specific catalytic process occurring during electrocatalysis, it is certain that a greater number of effective active sites in the catalyst leads to better catalytic activity. In the process of optimizing the performance of ZABs catalysts, atomic doping on carbon can lead to synergy and structural defects as well as adjustment of the electronic structure, coordination environment and catalyst structure. Therefore, the electrocatalytic effect of the catalyst can be improved by doping the carbon materials with specific atoms. Atomic doping can be categorized into single-atom doping and multi-atom doping, wherein the atoms can be either metal atoms or heteroatoms. Both single-atom doping and multi-atom doping can improve the electrocatalytic performance of the catalyst.

#### Atomic doping

Through compositional analysis and density functional theory calculation, Yu et al. established that N-doping can effectively improve the conductivity and oxygen absorption capacity of the catalyst; however, excessive N-doping causes a decrease in the reaction kinetics [45]. Synergistic effect and structural defects can be achieved by heteroatom doping.

#### Single-atom doping

**Fig. 3:**
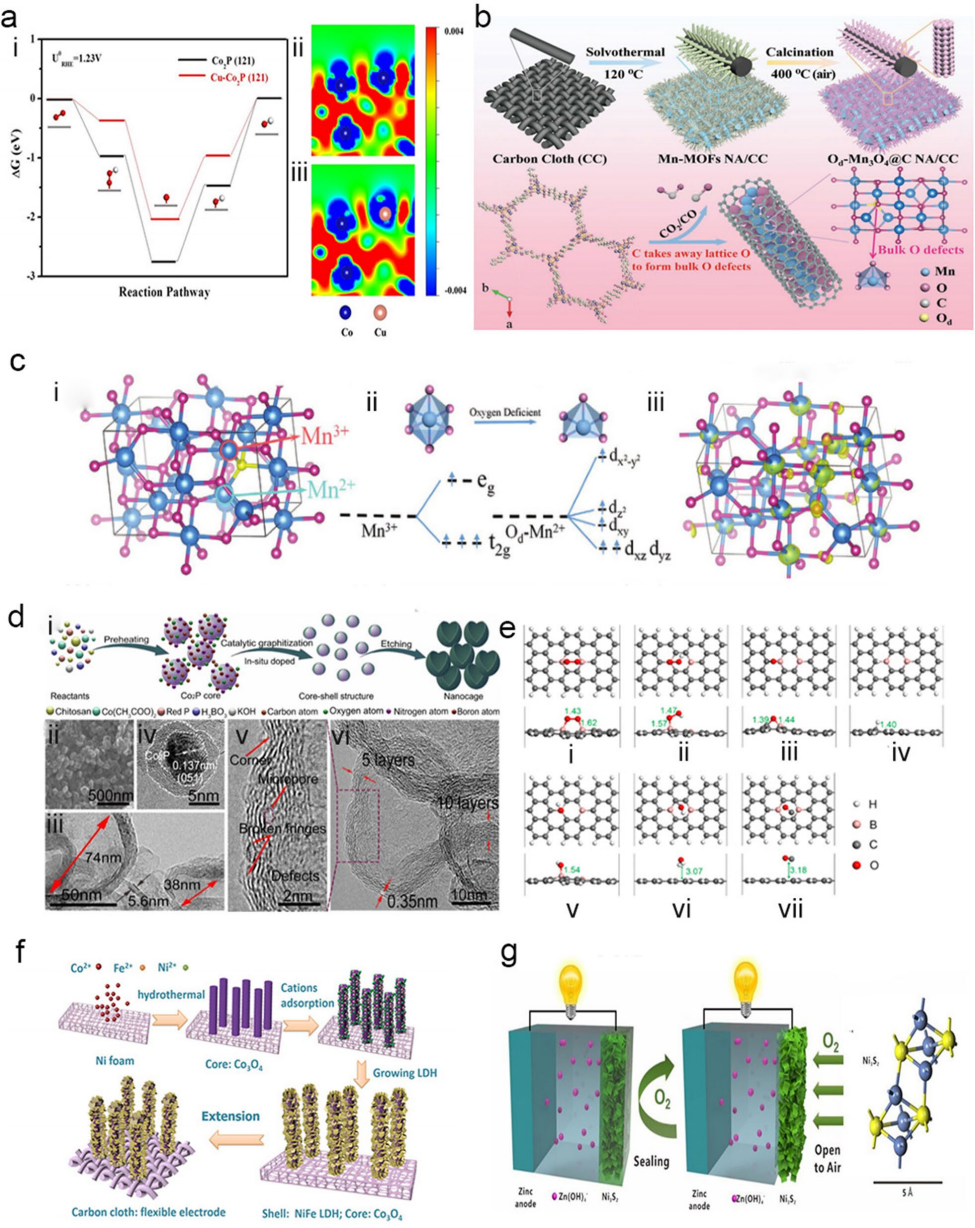
**a** (i) Free-energy diagram of the ORR over Co_2_P and Cu-doped Co_2_P surfaces. The different charge densities of (ii) Co_2_P (121) and (iii) Cu-doped Co_2_P (121). The blue and red regions separately indicate the depletion and accumulation of electrons [22]. **b** Illustration of the synthesis procedure of the Od-Mn_3_O_4_@CNA/CC nanostructure, and its formation mechanisms at atomic scale. The blue, pink, gray and yellow spheres represent the Mn, O, C and Od atoms, respectively [46]. **c** (i) Supercell model of Mn_3_O_4_. (ii) Mn–O octahedral and pyramidal crystal fields and the d-orbital splitting configurations. (iii) Electron density differences of Od-Mn_3_O_4_ (pink circle represents Mn^3+^, sky blue circle represents Mn^2+^) [47]. **d** Synthesis and morphological characterization of NB-CN [52]. (i) Illustration of the formation mechanism of the graphitic carbon nanocage. (ii) SEM image and (iii) TEM image of NB-CN. (iv) HR-TEM image of NB-CN before acid washing and (v) HR-TEM image of NB-CN. **e** Optimized adsorption structures of the ORR intermediates and CO on BGNR [54]: (i) O_2_, (ii) OOH, (iii) O, (iv) H, (v) OH, (vi) H_2_O and (vii) CO. **f** Schematic diagram of the synthetic process of the Co_3_O_4_@NiFe LDH hybrid nanowire arrays on Ni foam and flexible carbon cloth, respectively [24]. **g** Schematic illustration of Zn- Ni_3_S_2_ battery and zinc-air battery and structure diagram of Ni_3_S_2_ molecular, respectively [23]

Heteroatoms (N, P, S, etc.) and metals (Fe, Co, Mn, etc.) can be used for single-atom doping. Among them, N-doping is the most commonly used single-atom doping on carbon. N-doping can increase the electron transport efficiency and oxygen adsorption strength, as well as improve the reaction kinetics of the catalyst, resulting in defects and the exposure of more active sites. For example, Yu et al. demonstrated that N-doping can significantly improve the electronic conductivity and O_2_ adsorption capability of Co_3_O_4_ nanowires through experimental investigations and density functional theory (DFT) calculations [45]. Owing to the gap between the sheets, transition metals, such as Co [5], Ni, Mn [46, 47] (as shown in Fig. 3b, c), Fe and Cu, can be doped on active substrate materials, such as carbon materials (graphene, CNTs, etc.). It has also been confirmed that 2D heteroatoms exhibiting unique structures and physical and chemical properties, such as N, P, S [22, 48] and B [49–54] (as shown in Fig. 3d, e), can improve the electrochemical and electrocatalytic performances of the catalysts.

#### Multi-atom doping

Multi-atom doping includes both heteroatom co-doping (N-S, N-P, N-B, etc.), and heteroatom and metal co-doping [55] (Fe-Co–N [56], Mn-N [57], Fe–N [58], Co-Fe–N-P [12], Co-Fe–N [39], Co-Mn-N-P [59], Co–Cu-N [60, 61], Co–Cu-P, etc.). On the one hand, heteroatom doping can effectively generate synergistic defect effects in the catalyst, leading to a higher catalytic activity [62, 63]. On the other hand, it has been confirmed that co-doping with transition metal atoms and heteroatoms can effectively improve the oxygen reduction performance of the catalyst [64, 65]. In addition, there exists a synergistic effect between multi-metals and heteroatoms that increases the electrocatalytic activity of the catalyst. Multi-metals can improve the conductivity and oxidation state of the catalyst, thereby increasing the amount of charge transfer of the catalyst [66] and the electrocatalytic performance of the catalyst [60]. For example, Diao et al. established through DFT that Cu-doping can lead to more positive sites adjacent to Co and weaken the binding force between the surface active sites and the adsorbed intermediates, thereby increasing the mass and charge transfer rates and the exposure of active sites [61]. As shown in Fig. 3a, in Cu-doped Co_2_P, clear electron depletion occurs on the Co sites neighboring the Cu atoms, indicating that Cu-doping can change the electron distribution of Co_2_P [22].

#### Synergistic effect

A synergistic effect in catalysis can be defined as a significant enhancement in the catalytic activity when several elements (metals or non-metals) or compounds are combined, in comparison with when these elements or compounds are used on their own. The synergistic effect can assist in the regulation of the electronic structure of the catalyst substrate, enhancing the electrocatalytic activity [37, 67], and can produce a strong coordination to produce more active sites [56]. This collaboration can be classified into the following categories:

- Highly active metal/non-metal groups experience synergism with the conductive doped carbon/nitrogen substrates. One example of this category is metal-nitrogen-carbon (M–N-C) compounds [68–70], such as Co–N-C compounds. Co–N-C active sites can exist on the interface between Co and the N-doped carbon, which may facilitate the formation/deposition of O*. Moreover, it has been shown that the Co-N_*x*_ sites and the N embedded in the carbon matrix are active sites in non-noble metal ORR hybrid catalysts [68]. Other examples include transition metals (Co and Fe) on N-doped carbon [39, 71, 72], pyridine-N [58], graphitization-N [71], Co-azo species [71], N, P co-doped materials, layered N-doped heteroporous carbon nanofibers that possess excellent electron transport paths and a high specific surface area [10] and graphene nanocomposites. Graphene nanocomposites have been synthesized through the in situ hydrothermal growth of CoSe and nickel selenide nanoparticles on graphene nanosheets (GNs). The synergistic effect between the composite nanoparticles and graphene enhances the electrochemical performance of the catalyst [73, 74]. Most importantly, it has been proved that the strong coordination between the metal center and pyridine-N can promote the formation of pyridine-NM active sites, and the electron-rich pyridine-N can effectively accelerate the charge transfer to the metal center, thereby greatly improving the ORR activity [75, 76].
- The synergy between metals endows the catalysts with an alloying effect that adjusts the electronic structure of the system and optimizes the combination of oxygen [74, 77]. The synergistic effect of bimetallic active sites on oxygen electrocatalysis has previously been studied. For the NiCo_2_S_4_@g-C_3_N_4_-CNT integrated flexible electrode, electrons are transferred from the bimetallic Ni/Co active site to the abundant pyridine-N in g-C_3_N_4_ and cooperate with the coupled conductive CNT to promote reversible oxygen electrocatalysis. Theoretical calculations indicate that the pyridine metal-N species (Ni, Co-N_2_) has a unique co-activation effect on the bimetallic Ni/Co atom. It reduces its d-band center and facilitates the adsorption/desorption of oxygen intermediates, thereby accelerating the reaction kinetics. In a Co-doped Fe-O_4_N@NC nanosheet array, the metal center can generate a strong coordination effect with the pyridine-N, and the Fe and N co-doping significantly promotes the formation of a large number of pyridine-N-M active sites in the ORR [78–81]. In hybridized porous Co_3_O_4_ anchored on MnO_2_, Co and Mn generate a coupling effect, thereby accelerating the electron transport rate, forming a buffer zone and accelerating the separation of the catalyst surface products [82]. Another example is Cu and Co-modified N-doped GNs with Co nanoparticles [60]. Furthermore, the excellent electrocatalytic activity of Co_2_P@CNF can be explained by the strong interaction between the Co_2_P nanocrystals and the porous carbon coating co-doped with CoNx and N and P, resulting in an enhanced interfacial charge transport and regulation of the Co_2_P electrocatalytic activity [83]. A new 2D MoSe_2_-Ni(OH)_2_ material has also been prepared through a simple one-step hydrothermal synthesis. The 2D MoSe_2_-Ni(OH)_2_ nanohybrid, with a unique vertical orientation nanosheet structure, provides a large amount of electroactive specific surface area, shortening the diffusion length of the electrolyte ions and thereby improving the electrochemical reaction kinetics [84].

#### Structural defects

Defects, including lattice distortion, broken stripes and edge sites at the corners, are considered to have a positive effect on catalytic activity. It has been established that defects at the edges of DG (defective graphene) can reduce the free-energy changes of ORR and OER, thereby improving the catalytic activity and conductivity of DG [85]. For example, the introduction of P atoms into the N-doped carbon matrix can effectively produce a synergistic defect effect and N-P structure, thereby optimizing the catalytic performance in the OER and ORR [12]. Meanwhile, H_2_ [86] and Ar plasma etching can also be used to form materials with rich defect structures.

#### Adjusting the electronic structure and coordination environment

Regarding the electronic structure of the catalyst, it has been widely estimated that the surface charge distribution of the catalyst can be adjusted by introducing defects, such as doped heterometallic cations. The defects, including oxygen vacancies (VO) [77], can increase the number of catalytic active sites or provide the catalyst with new catalytic activity [87]. VO can be achieved by Ar plasma etching [88]. In alkaline media, an efficient ORR catalyst should be able to completely reduce oxygen to hydroxide through the four-electron reduction process, whereas a weaker ORR catalyst terminates the reaction sequence in the middle of the two-electron transfer process. For example, Lian et al. proved that a 3D orbital configuration of the metal center promotes the ORR by adjusting the oxidation state and electronic state of the metal center. Additionally, local coordination can further accelerate the conversion rate of the target redox substances [10]. Co atoms also have excellent activity owing to their different possible valence states. Co^2+^ and Co^3+^ occupy the tetrahedral and octahedral sites of Co_3_O_4_, respectively, which helps in promoting electron transfer in the OER [11]. Furthermore, the deposition of ultrathin NiFe-layered double hydroxides (NiFe LDHs) on the surface of Co_3_O_4_ can adjust the surface chemical valence of Co, Ni and Fe by changing the electron donor and/or electron absorption effect, resulting in the balance and optimization of the ORR and OER performance [89], as shown in Fig. 3f.

#### Increasing the number of pore structures

Nanostructured materials have rich pore structures and large numbers of mesoporous channels, which play a vital role in modifying oxygen transmission channels [11], extending the capture time of oxygen molecules and enhancing their interaction with the catalytic active sites. Some nanostructured materials exhibit a 2D/3D structure [90], which has a larger specific surface area and increases the number of three-phase interfaces. By rational design, a nanostructured porous material with a large surface area and high conductivity can be constructed to ensure that more nanoparticles are in direct contact with the electrolyte, resulting in a lower resistance between the active material and the electrolyte [83]. A rich pore structure can accelerate charge transfer and diffusion between charges and increase mass transfer, thereby improving the performance of the catalyst [11, 39]. The surfaces of nanostructured materials have a stable low-refractive-index and can exhibit oxygen adsorption energies similar to the surface of Pt [91]. This feature is part of the conditions required for catalyst’s highly efficient catalytic performance. Nanostructured materials that can be used as the air cathode of ZABs include Ni_3_C/NC nanosheets (2D), Ni_3_S_2_/Ni nanosheet arrays [1] (as shown in Fig. 3g), uniform porous Co_3_O_4_ nanoparticles/nanosheets [11, 45], N-doped CNTs (usually used as an active substrate), hollow NiCo_2_O_4_ nanospheres, N-doped CNTs [92], and MoSe_2_/G nano-hybrids [93].

#### Adjusting the catalyst structure

A catalyst with a reasonable structure is required to catalyze a reaction. Transition metal oxides with a spinel structure can help reduce overpotentials, thereby improving the energy conversion efficiency of the catalyst [82, 94]. The 2D/3D structure of the catalyst can result in an excellent catalytic activity owing to the enlarged contact area between the active site and the electrolyte. For example, Co-doped NiO nanoporous flowers, in which the synergistic effect between the 2D hexagonal frame and a large number of nanopores on the side of the nanosheet increases the number of effective catalytic active sites for O_2_ adsorption/diffusion [5]; and the unique 3D-layered Co/Co-NC system structure that can provide an efficient number of active sites [29]. Other examples include a new graphene hydrogen/B-doped quantum dot composite material that has a unique 3D structure, high porosity and large specific surface area, which exhibits an abundance of catalytically active sites and enhanced electrolyte mass transport and ion diffusion [95], and a 2D Co-MOF that is grown directly on CC providing the 3D Co-MOF growth sufficient space to form a layered 3D-on-2D MOF system structure. In comparison with an exclusively 3D or 2D MOF, more catalytically active sites are exposed [24]. Furthermore, the 3D framework facilitates full penetration of the electrolyte and promotes electron transport in the porous network structure [41]. In addition, miniaturizing the volume of the catalytic material can also improve catalytic efficiency. For example, at the atomic scale, an ultrathin CoO_x_ layer effectively accelerates electron conduction and provides abundant active sites. This is owing to the introduction of Co oxidation in the nanosheets that can increase the number of exposed active centers [23]. Another example includes a 2D La(OH)_3_-graphene nanohybrid, prepared by a simple and economical solvothermal reduction technique, which is electrostatically anchored on 2D GNs to prevent the aggregation of lanthanum hydroxide and provide several electroactive centers for the reaction [96].

## Conclusions and future prospects

Although the current research on ZABs has made some substantial progress, there are still extensive challenges, including the development of new methods for synthesizing self-supporting flexible cathodes, exploring electrocatalytic mechanisms and identifying suitable materials to synthesize flexible cathodes with excellent catalytic performance. As one of the important catalyst materials for ZABs, carbon-based catalysts have attracted significant attention owing to their large specific surface area, abundant active centers and good electrical conductivity. However, carbon materials still suffer from some disadvantages, such as uncertain toxicity of CNTs, higher cost than other flexible electrocatalyst materials and poor repeatability on different substrates; carbon materials derived from natural biomaterials have relatively poor flexibility and conductivity, which limits the performance of the wearable electronic devices in which they are applied. An extensive study regarding the catalyst microstructure and effect of atomic doping, as well as the internal relationship between the electronic distribution of the catalyst and its electrocatalytic oxygen reduction performance, is expected to play a vital role in effectively identifying the actual role of metal ions, N, S and P, and other doping elements on the active sites and deepen the understanding of the carbon catalyst electrocatalysis ORR process. We expected to contribute to the development of affordable, high-performance carbon-based non-noble metal ORR catalysts. A carbon-based flexible electronic products have been used to detect the human pulse and breathing rate [97]. It is believed that more multifunctional flexible electronic devices based on carbon-based flexible ZAB catalysts will be commercialized in the future.

## Data Availability

Not applicable. All pictures in the article have been cited.

## Abbreviations

ZABs: Zinc-air batteries
ORR: Oxygen reduction reaction
OER: Oxygen evolution reaction
CC: Carbon cloth
PEO: Polyethylene oxide
PAM: Polyacrylamide
PAA: Polyacrylic acid
CNTs: Carbon nanotubes
TMP: Transition metal phosphides
CFP: Carbon fiber paper
DFT: Density functional theory
DG: Defective graphene
VO: Oxygen vacancies
NiFe LDHs: NiFe-layered double hydroxides

## References

[1] Huang ZD, Li XL, Yang Q, Ma LT, Mo FN, Liang GJ, Wang DH, Liu ZX, Li HF, Zhi CY (2019). Ni_3_S_2_/Ni nanosheet arrays for high-performance flexible zinc hybrid batteries with evident two-stage charge and discharge processes. J Mater Chem A.

[2] Chen S, Shu XX, Wang HS, Zhang JT (2019). Thermally driven phase transition of manganese oxide on carbon cloth for enhancing the performance of flexible all-solid-state zinc-air batteries. J Mater Chem A.

[3] Peng L, Shen J, Zheng X, Xiang R, Deng M, Mao Z, Feng Z, Zhang L, Li L, Wei Z (2019). Rationally design of monometallic NiO-Ni_3_S_2_/NF heteronanosheets as bifunctional electrocatalysts for overall water splitting. J Catal.

[4] Schmitt T, Arlt T, Manke I, Latz A, Horstmann B (2019). Zinc electrode shape-change in secondary air batteries: A 2D modeling approach. J Power Sources.

[5] Qian JM, Guo XS, Wang TT, Liu PT, Zhang H, Gao DQ (2019). Bifunctional porous Co-doped NiO nanoflowers electrocatalysts for rechargeable zinc-air batteries. Appl Catal B-Environ.

[6] Guan Q, Li YP, Bi XX, Yang J, Zhou JW, Li XL, Cheng JL, Wang ZP, Wang B, Lu J (2019). Dendrite-free flexible fiber-shaped zn battery with long cycle life in water and air. Adv Energy Mater.

[7] Genshaw MA, Damjanovic A, Bockris JOM (1967). Role of hydrogen peroxide in oxygen reduction at rhodium electrodes. J Phys Chem.

[8] Ramaswamy N, Mukerjee S (2011). Influence of inner- and outer-sphere electron transfer mechanisms during electrocatalysis of oxygen reduction in alkaline media. J Phys Chem C.

[9] Gojković SL, Gupta S, Savinell RF (1999). Heat-treated iron(III) tetramethoxyphenyl porphyrin chloride supported on high-area carbon as an electrocatalyst for oxygen reduction: part II. Kinetics of oxygen reduction. J Electroanal Chem.

[10] Yan JC, Tang ZM, Li BX, Bi D, Lai QX, Liang YY (2019). In situ ZnO-activated hierarchical porous carbon nanofibers as self-standing electrodes for flexible Zn–Air batteries. ACS Sustain Chem Eng.

[11] Wang PC, Wan L, Lin YQ, Wang BG (2019) Construction of mass-transfer channel in air electrode with bifunctional catalyst for rechargeable zinc-air battery. Electrochim Acta 320:134564

[12] Ma Y, Gan L, Li D, Gao Y, Yang X, Wang K, Lu S, Wu H, Ding S, Xiao C (2019) Rational modulation of N, P co-doped carbon nanotubes encapsulating Co3Fe7 alloy as bifunctional oxygen electrocatalysts for Zinc-Air batteries. J Power Sources 441:227177

[13] Tang K, Yuan C, Xiong Y, Hu H, Wu M (2020). Inverse-opal-structured hybrids of N, S-codoped-carbon-confined Co_9_S_8_ nanoparticles as bifunctional oxygen electrocatalyst for on-chip all-solid-state rechargeable Zn-air batteries. Appl Catal B Environ.

[14] Zhu YH, Yang XY, Liu T, Zhang XB (2020) Flexible 1D batteries: recent progress and prospects. Adv Mater 32(5):1901961 10.1002/adma.201901961 31328846

[15] Shi F, Zhu X, Yang W (2020) Micro-nanostructural designs of bifunctional electrocatalysts for metal-air batteries. Chin J Catal 41(3):390–403

[16] Hu H, Xin J, Hu H, Wang X, Kong Y (2015). Metal-free graphene-based catalyst-Insight into the catalytic activity: a short review. Appl Catal A Gen.

[17] Chen S, Chen S, Zhang JT (2019). Thermal sugar bubbling preparation of N-doped porous carbon for high-performance solid-state Zn–Air batteries. Batteries Supercaps.

[18] Xiang Y, Yang T, Tong K, Fu T, Tang Y, Liu F, Xiong Z, Si Y, Guo C (2020). Constructing flexible and self-standing electrocatalyst for oxygen reduction reaction by in situ doping nitrogen atoms into carbon cloth. Appl Surf Sci.

[19] Wang J, Li K, Zhong HX, Xu D, Wang ZL, Jiang Z, Wu ZJ, Zhang XB (2015). Synergistic effect between metal-nitrogen-carbon sheets and NiO nanoparticles for enhanced electrochemical water-oxidation performance. Angew Chem Int Ed.

[20] Zhu YP, Ma TY, Jaroniec M, Qiao SZ (2017). Self-templating synthesis of hollow Co_3_O_4_ microtube arrays for highly efficient water electrolysis. Angew Chem Int Ed.

[21] Kou TY, Smart T, Yao B, Chen I, Thota D, Ping Y, Li Y (2018). Theoretical and experimental insight into the effect of nitrogen doping on hydrogen evolution activity of Ni_3_S_2_ in alkaline medium. Adv Energy Mater.

[22] Diao LC, Yang T, Chen B, Zhang B, Zhao NQ, Shi CS, Liu EZ, Ma LY, He CN (2019). Electronic reconfiguration of Co_2_P induced by Cu doping enhancing oxygen reduction reaction activity in zinc-air batteries. J Mater Chem A.

[23] Zhou TP, Xu WF, Zhang N, Du ZY, Zhong CG, Yan WS, Ju HX, Chu WS, Jiang H, Wu CZ, Xie Y (2019). Ultrathin cobalt oxide layers as electrocatalysts for high-performance flexible Zn-air batteries. Adv Mater.

[24] Zhong YT, Pan ZH, Wang XS, Yang J, Qiu YC, Xu SY, Lu YT, Huang QM, Li WS (2019). Hierarchical Co_3_O_4_ nano-micro arrays featuring superior activity as cathode in a flexible and rechargeable zinc-air battery. Adv Sci.

[25] Meng F, Zhong H, Bao D, Yan J, Zhang X (2016). In situ coupling of strung Co_4_N and intertwined N–C fibers toward free-standing bifunctional cathode for robust, efficient, and flexible Zn–Air batteries. J Am Chem Soc.

[26] Chen X, Liu B, Zhong C, Liu Z, Liu J, Ma L, Deng YD, Han XP, Wu TP, Hu WB, Lu J (2017) Ultrathin Co_3_O_4_ layers with large contact area on carbon fibers as high-performance electrode for flexible zinc-air battery integrated with flexible display. Adv Energy Mater 7(18):1700779

[27] Pan Z, Yang J, Zang W, Kou Z, Wang C, Ding X, Guan C, Xiong T, Chen H, Zhang Q, Zhong Y, Liu M, Xing L, Qiu Y, Li W, Yan C, Zhang Y, Wang J (2019). All-solid-state sponge-like squeezable zinc-air battery. Energy Storage Mater.

[28] Jiang Y, Deng YP, Liang RL, Fu J, Luo D, Liu GH, Li JD, Zhang Z, Hu YF, Chen ZW (2019). Multidimensional ordered bifunctional air electrode enables flash reactants shuttling for high-energy flexible Zn–Air batteries. Adv Energy Mater.

[29] Yu P, Wang L, Sun FF, Xie Y, Liu X, Ma JY, Wang XW, Tian CG, Li JH, Fu HG (2019). Co nanoislands rooted on Co–N–C nanosheets as efficient oxygen electrocatalyst for Zn-air batteries. Adv Mater.

[30] Zheng Y, Liu J, Liang J, Jaroniec M, Qiao S (2012) Graphitic carbon nitride materials: controllable synthesis and applications in fuel cells and photocatalysis. Energy Environ Sci (5):6717–6731

[31] Huijun Yu, Lu S, Tong B, Run S, Geoffrey I (2016). Nitrogen-doped porous carbon nanosheets templated from g-C_3_N_4_ as metal-free electrocatalysts for efficient oxygen reduction reaction. Adv Mater.

[32] Yang Z, Wang Y, Zhu M, Li Z, Chen W, Wei W, Yuan T, Qu Y, Xu Q, Zhao C, Wang X, Li P, Li Y, Wu Y, Li Y (2019). Boosting oxygen reduction catalysis with Fe–N4 sites decorated porous carbons toward fuel cells. ACS Catal.

[33] Pan F, Jin J, Fu X, Liu Q, Zhang J (2013). Advanced oxygen reduction electrocatalyst based on nitrogen-doped graphene derived from edible sugar and urea. Acs Appl Mater Interfaces.

[34] Wen Z, Ci S, Hou Y, Chen J (2014). Facile one-pot, one-step synthesis of a carbon nanoarchitecture for an advanced multifunctonal electrocatalyst. Angew Chem Int Ed.

[35] Deng Y, Tian X, Chi B, Wang Q, Ni W, Gao Y, Liu Z, Luo J, Lin C, Ling L, Cheng F (2020). Hierarchically open-porous carbon networks enriched with exclusive Fe–Nx active sites as efficient oxygen reduction catalysts towards acidic H2–O2 PEM fuel cell and alkaline Zn–air battery. Chem Eng J.

[36] Ma TY, Ran JR, Dai S, Jaroniec M, Qiao SZ (2015). Phosphorus-doped graphitic carbon nitrides grown in situ on carbon-fiber paper: flexible and reversible oxygen electrodes. Angew Chem Int Ed.

[37] Xu YY, Deng PL, Chen GD, Chen JX, Yan Y, Qi K, Liu HF, Xia BY (2020) 2D nitrogen-doped carbon nanotubes/graphene hybrid as bifunctional oxygen electrocatalyst for long-life rechargeable Zn-air batteries. Adv Funct Mater 30(6):1906081

[38] Guo C, Li Y, Li Z, Liu Y, Si Y, Luo Z (2020) Nanochannel-controlled synthesis of ultrahigh nitrogen-doping efficiency on mesoporous Fe/N/C catalysts for oxygen reduction reaction. Nanoscale Res Lett 15(21) 10.1186/s11671-020-3254-x PMC6987278 31993836

[39] Jin Q, Bowen R, Jianp C, Cui H, Wang C (2019) A facile method to conduct 3D self-supporting Co-FeCo/N-doped graphenelike carbon bifunctional electrocatalysts for flexible solid-state zinc air battery. Appl Catal B Environ 256:117887

[40] Zhang HM, Zhao Y, Zhang YJ, Zhang MH, Cheng MS, Yu JL, Liu HC, Ji MW, Zhu CZ, Xu J (2019) Fe_3_O_4_ encapsulated in porous carbon nanobowls as efficient oxygen reduction reaction catalyst for Zn-air batteries. Chem Eng J 375:122058

[41] Huang ZX, Qin XP, Li GZ, Yao WC, Liu J, Wang NG, Ithisuphalap K, Wu G, Shao MH, Shi ZC (2019). Co_3_O_4_ nanoparticles anchored on nitrogen-doped partially exfoliated multiwall carbon nanotubes as an enhanced oxygen electrocatalyst for the rechargeable and flexible solid-state Zn-air battery. ACS Appl Energy Mater.

[42] Xu YF, Zhang Y, Guo ZY, Ren J, Wang YG, Peng HS (2015). Flexible, stretchable, and rechargeable fiber-shaped zinc-air battery based on cross-stacked carbon nanotube sheets. Angew Chem Int Ed.

[43] Lee D, Lee H, GwonO h, Kwon O, Jeong HY, Kim G, Lee S-Y (2019) Monolithic heteronanomat paper air cathodes toward origamifoldable/rechargeable Zn–Air batteries. J Mater Chem A 7(42):24231-24238

[44] Liu Q, Wang YB, Dai LM, Yao JN (2016). Scalable fabrication of nanoporous carbon fiber films as bifunctional catalytic electrodes for flexible Zn-air batteries. Adv Mater.

[45] Yu MH, Wang ZK, Hou C, Wang ZL, Liang CL, Zhao CY, Tong YX, Lu XH, Yang SH (2017). Nitrogen-Doped Co_3_O_4_ mesoporous nanowire arrays as an additive-free air-cathode for flexible solid-state zinc-air batteries. Adv Mater.

[46] Tan Q, Li X, Zhang B, Chen X, Tian Y, Wan H, Zhang L, Miao L, Wang C, Gan Y, Jiang J, Wang Y, Wang H (2020). Valence engineering via in situ carbon reduction on octahedron sites Mn_3_O_4_ for ultra-long cycle life aqueous Zn-ion battery. Adv Energy Mater.

[47] Zhang L, Xie XY, Wang H, Ji L, Zhang Y, Chen H, Li T, Luo Y, Cui G, Sun X (2019). Boosting electrocatalytic N_2_ reduction by MnO_2_ with oxygen vacancies. Chem Commun (Camb).

[48] Shinde SS, Yu JY, Song JW, Nam YH, Kim DH, Lee JH (2017). Highly active and durable carbon nitride fibers as metal-free bifunctional oxygen electrodes for flexible Zn-air batteries. Nanoscale Horizons.

[49] Zhang JT, Dai LM (2015). Heteroatom-doped graphitic carbon catalysts for efficient electrocatalysis of oxygen reduction reaction. ACS Catal.

[50] Agnoli S, Favaro M (2016). Doping graphene with boron: a review of synthesis methods, physicochemical characterization, and emerging applications. J Mater Chem A.

[51] Wang QC, Ji YJ, Lei YP, Wang YB, Wang YD, Li YY, Wang SY (2018). Pyridinic-N-dominated doped defective graphene as a superior oxygen electrocatalyst for ultrahigh-energy-density Zn-air batteries. ACS Energy Lett.

[52] Lu ZY, Wang J, Huang SF, Hou YL, Li YG, Zhao YP, Mu SC, Zhang JJ, Zhao YF (2017). N, B-codoped defect-rich graphitic carbon nanocages as high performance multifunctional electrocatalysts. Nano Energy.

[53] Favaro M, Ferrighi L, Fazio G, Colazzo L, Di Vaentin C, Durante C, Sedona F, Gennaro A, Agnoli S, Granozzi G (2015). Single and multiple doping in graphene quantum dots: unraveling the origin of selectivity in the oxygen reduction reaction. ACS Catal.

[54] Wang L, Dong HL, Guo ZY, Zhang LL, Hou TJ, Li YY (2016). Potential application of novel boron-doped graphene nanoribbon as oxygen reduction reaction catalyst. J Phys Chem C.

[55] Guan C, Sumboja A, Zang W, Qian Y, Zhang H, Liu X, Liu Z, Zhao D, Pennycook SJ, Wang J (2019). Decorating Co/CoNx nanoparticles in nitrogen-doped carbon nanoarrays for flexible and rechargeable zinc-air batteries. Energy Storage Mater.

[56] Qiucheng X, Hao J, Yuhang L, Da L, Yanjie H, Chunzhong L (2019). In-situ enriching active sites on co-doped Fe-Co_4_N@N-C nanosheet array as air cathode for flexible rechargeable Zn-air batteries. Appl Catal B Environ.

[57] Li J, Chen M, Cullen DA, Hwang S, Wang M, Li B, Liu K, Karakalos S, Lucero M, Zhang H, Lei C, Xu H, Sterbinsky GE, Feng Z, Su D, More KL, Wang G, Wang Z, Wu G (2018) Atomically dispersed manganese catalysts for oxygen reduction in proton-exchange membrane fuel cells. Nat Catal 1:935–945

[58] Luo X, Wei X, Wang H, Gu W, Kaneko T, Yoshida Y, Zhao X, Zhu C (2020) Secondary-atom-doping enables robust Fe–N–C single-atom catalysts with enhanced oxygen reduction reaction. Nano-Micro Lett 12:163 10.1007/s40820-020-00502-5 PMC7770947 34138162

[59] Yang S, He Q, Wang C, Jiang H, Wu C, Zhang Y, Zhou T, Zhou Y, Song L (2018). Confined bimetallic phosphide within P, N co-doped carbon layers towards boosted bifunctional oxygen catalysis. J Mater Chem A.

[60] Liu PT, Hu YT, Liu XK, Wang TT, Xi PX, Xi SB, Gao DQ, Wang J (2019) Cu and Co nanoparticle Co-decorated N-doped graphene nanosheets: a high efficiency bifunctional electrocatalyst for rechargeable Zn-air batteries. J Mater Chem A 7(20):12851-12858

[61] Diao L, Yang T, Chen B, Zhang B, Zhao N, Shi C, Liu E, Ma L, He C (2019). Electronic reconfiguration of Co_2_P induced by Cu doping enhancing oxygen reduction reaction activity in zinc–air batteries. J Mater Chem A.

[62] Zhang J, Zhao Z, Xia Z, Dai L (2015) A metal-free bifunctional electrocatalyst for oxygen reduction and oxygen evolution reactions. Nat Nanotechnol 10:444–452 10.1038/nnano.2015.48 25849787

[63] Yi J-D, Xu R, Wu Q, Zhang T, Zang K-T, Luo J, Liang Y-L, Huang Y-B, Cao R (2018). Atomically dispersed iron-nitrogen active sites within porphyrinic triazine-based frameworks for oxygen reduction reaction in both alkaline and acidic media. ACS Energy Lett.

[64] Zan Y, Zhang Z, Liu H, Dou M, Wang F (2017). Nitrogen and phosphorus co-doped hierarchically porous carbons derived from cattle bones as efficient metal-free electrocatalysts for the oxygen reduction reaction. J Mater Chem A.

[65] Chai G-L, Qiu K, Qiao M, Titirici M-M, Shang C, Guo Z (2017). Active sites engineering leads to exceptional ORR and OER bifunctionality in P, N Co-doped graphene frameworks. Energy Environ Sci.

[66] Gao J, Wang J, Zhou L, Cai X, Zhan D, Hou M, Lai L (2019). Co2P@N, P-codoped carbon nanofiber as a free-standing air electrode for Zn–Air batteries: synergy effects of CoNx satellite shells. ACS Appl Mater Interfaces.

[67] Cao ZH, Haibo, Wu M (2019) Planar all-solid-state rechargeable Zn–air batteries for compact wearable energy storage. J Mater Chem A 7(29):17581–17593

[68] Pei ZX, Huang Y, Tang ZJ, Ma LT, Liu ZX, Xue Q, Wang ZF, Li HF, Chen Y, Zhi CY (2019). Enabling highly efficient, flexible and rechargeable quasi-solid-state zn-air batteries via catalyst engineering and electrolyte functionalization. Energy Storage Mater.

[69] Sun M, Davenport D, Liu HJ, Qu JH, Elimelech M, Li JH (2018). Highly efficient and sustainable non-precious-metal Fe–N–C electrocatalysts for the oxygen reduction reaction. J Mater Chem A.

[70] Guo CZ, Li YR, Liao WL, Liu Y, Li ZB, Sun LT, Chen CG, Zhang J, Si YJ, Li L (2018). Boosting the oxygen reduction activity of a three-dimensional network Co–N–C electrocatalyst via space-confined control of nitrogen-doping efficiency and the molecular-level coordination effect. J Mater Chem A.

[71] Lee D, Lee H, Gwon O, Kwon O, Jeong HY, Kim G, Lee SY (2019). Monolithic heteronanomat paper air cathodes toward origami-foldable/rechargeable Zn-air batteries. J Mater Chem A.

[72] Su CY, Cheng H, Li W, Liu ZQ, Li N, Hou ZF, Bai FQ, Zhang HX, Ma TY (2017). Atomic modulation of FeCo-nitrogen-carbon bifunctional oxygen electrodes for rechargeable and flexible all-solid-state zinc-air battery. Adv Energy Mater.

[73] Murugadoss V, Wang N, Tadakamalla S, Wang B, Guo Z, Angaiah S (2017) In-situ grown cobalt selenide/graphene nanocomposites counter electrode for enhanced dye-sensitized solar cell performance. J Mater Chem A 5(28):14583–14594

[74] Kirubasankar B, Murugadoss V, Lin J, Dong MY, Liu H, Zhang JX, Li TX, Wang N, Guo ZH, Angaiah S (2018) In situ grown nickel selenide on graphene nanohybrid electrodes for high energy density asymmetric supercapacitors. Nanoscale 10(43):20414–20425 10.1039/c8nr06345a 30377681

[75] Liang Y, Wang H, Diao P, Chang W, Hong G, Li Y, Gong M, Xie L, Zhou J, Wang J (2012). Oxygen reduction electrocatalyst based on strongly coupled cobalt oxide nanocrystals and carbon nanotubes. J Am Chem Soc.

[76] Xue-Rui W, Jie-Yu L, Zi-Wei L, Wei-Chao W, Jun L, Xiao-Peng H, Xi-Wen D, Shi-Zhang Q, Jing Y (2018). Identifying the key role of Pyridinic-N–Co bonding in synergistic electrocatalysis for reversible ORR/OER. Adv Mater.

[77] Luo M, Zhao Z, Zhang Y, Sun Y, Xing Y, Lv F, Yang Y, Zhang X, Hwang S, Qin Y, Ma JY, Lin F, Su D, Lu G, Guo S (2019). PdMo bimetallene for oxygen reduction catalysis. Nature.

[78] Peera SG, Arunchander A, Sahu AK (2016). Cumulative effect of transition metals on nitrogen and fluorine co-doped graphite nanofibers: an efficient and highly durable non-precious metal catalyst for the oxygen reduction reaction. Nanoscale.

[79] Yang L, Shi L, Wang D, Lv YL, Cao DP (2018). Single-atom cobalt electrocatalysts for foldable solid-state Zn-air battery. Nano Energy.

[80] Meng FL, Wang ZL, Zhong HX, Wang J, Yan JM, Zhang XB (2016). Reactive multifunctional template-induced preparation of Fe–N-doped mesoporous carbon microspheres towards highly efficient electrocatalysts for oxygen reduction. Adv Mater.

[81] Zhao J, Fu N, Liu R (2018). Graphite-wrapped Fe core shell nanoparticles anchored on graphene as pH-universal electrocatalyst for oxygen reduction reaction. ACS Appl Mater Interfaces.

[82] Xu NN, Zhang YX, Wang M, Fan XJ, Zhang T, Peng LW, Zhou XD, Qiao JL (2019) High-performing rechargeable/flexible zinc-air batteries by coordinated hierarchical Bi-metallic electrocatalyst and heterostructure anion exchange membrane. Nano Energy 65:104021

[83] Gao JC, Wang JM, Zhou LJ, Cai XY, Zhan D, Hou MZ, Lai LF (2019). Co_2_P@N, P-codoped carbon nanofiber as a free-standing air electrode for Zn-air batteries: synergy effects of CoNx satellite shells. ACS Appl Mater Interfaces.

[84] Balakrishnan K, Angaiah S, Murugadoss V, Subasri A (2018) 2D MoSe_2_-Ni(OH)_2_ nanohybrid as an efficient electrode material with high rate capability for asymmetric supercapacitor applications. Chem Eng J 355:881-890

[85] Liu LN, Wang Y, Yan F, Zhu CL, Geng B, Chen YJ, Chou SL (2020) Cobalt-encapsulated nitrogen-doped carbon nanotube arrays for flexible zinc-air batteries. Small Methods 4(1):1900571

[86] Wang HF, Tang C, Wang B, Li BQ, Cui XY, Zhang Q (2018). Defect-rich carbon fiber electrocatalysts with porous graphene skin for flexible solid-state zinc-air batteries. Energy Storage Mater.

[87] Wang YF, Han P, Lv XM, Zhang LJ, Zheng GF (2018). Defect and interface engineering for aqueous electrocatalytic CO_2_ reduction. Joule.

[88] Ma LT, Chen SM, Pei ZX, Li HF, Wang ZF, Liu ZX, Tang ZJ, Zapien JA, Zhi CY (2018). Flexible waterproof rechargeable hybrid zinc batteries initiated by multifunctional oxygen vacancies-rich cobalt oxide. ACS Nano.

[89] Guo XL, Hu XL, Wu D, Jing C, Liu W, Ren ZL, Zhao QN, Jiang XP, Xu CH, Zhang YX, Hu N (2019). Tuning the bifunctional oxygen electrocatalytic properties of core-shell Co_3_O_4_@NiFe LDH catalysts for Zn-air batteries: effects of interfacial cation valences. ACS Appl Mater Interfaces.

[90] Hao J, Zhang G, Zheng Y, Luo W, Jin C, Wang R, Wang Z, Zhen W (2019) Controlled synthesis of Ni_3_C/nitrogen-doped carbon nanoflakes for efficient oxygen evolution. Electrochimica Acta 320:134631

[91] Yuan Y, Wang J, Adimi S, Shen H, Thomas T, Ma R, Attfield JP, Yang M (2020) Zirconium nitride catalysts surpass platinum for oxygen reduction. Nat Mater 19:282–286 10.1038/s41563-019-0535-9 31740792

[92] Ge HY, Li GD, Zheng T, Wang FB, Shao MJ, Liu HY, Meng XG (2019). Hollow NiCo_2_O_4_ nanospheres supported on N-doped carbon nanowebs as efficient bifunctional catalyst for rechargeable and flexible Zn-air batteries. Electrochim Acta.

[93] Kirubasankar B, Vijayan S, Angaiah S (2019) Sonochemical synthesis of 2D-2D MoSe_2_/Graphene nanohybrid electrode material for asymmetric supercapacitors. Sustain Energy Fuels 3(2):467–477

[94] Lee JS, Kim ST, Cao R, Choi NS, Liu M, Lee KT, Cho J (2011). Metal-Air Batteries with High Energy Density: Li-Air versus Zn-Air. Adv Energy Mater.

[95] Tam TV, Kang SG, Kim MH, Lee SG, Hur SH, Chung JS, Choi WM (2019). Novel graphene hydrogel/B-doped graphene quantum dots composites as trifunctional electrocatalysts for Zn-Air batteries and overall water splitting. Adv Energy Mater.

[96] Asa B, Kb A, Ern B, Vd B, As A (2018). Development of 2D La(OH)_3_/graphene nanohybrid by a facile solvothermal reduction process for high-performance supercapacitors. Electrochim Acta.

[97] Wang CY, Xia KL, Wang HM, Liang XP, Yin Z, Zhang YY (2019) Advanced Carbon for Flexible and Wearable Electronics. Advanced Materials 31(9):1801072 10.1002/adma.201801072 30300444

[98] Liu L, Zhang X, Yan F, Geng B, Zhu C, Chen Y (2020). Self-supported N-doped CNT arrays for flexible Zn–air batteries. J Mater Chem A.

